# Microbial Musings – May 2021

**DOI:** 10.1099/mic.0.001069

**Published:** 2021-06-08

**Authors:** Gavin H. Thomas

**Affiliations:** ^1^​ Department of Biology, University of York, York, UK

We start this month with our latest Microbe Profile, which describes the species *Aeromonas salmonicida *, a Gram-negative γ-proteobacterium that is known best as a fish pathogen [1]. The article, written by Steve Charette from the IBIS (@ibis_laval), L’Université Laval, Canada, outlines the features of the archetypal member of this species, a psychrophile that causes the disease furunculosis in salmon. He quickly goes on to reveal the diversity of subspecies uncovered through genomics, including mesophilic representatives that cause infection in other animals, including humans. Plasmid-encoded type III secretions systems (T3SSs) are key virulence factors and required for fish infection, as revealed in work that was published in *Microbiology* in 2006 from Andrew Dacanay and colleagues at the Institute for Marine Biosciences, Halifax, Nova Scotia, Canada [2]. As infections by these bacteria impact on fish aquaculture and antimicrobial resistance is also increasing, new routes to treatments are being investigated and the use of bacteriophage, although not unique as an approach, appears to show promise [3].

Bacteriophage are also the topic for the first paper published in our new Microbial Evolution topic area, from author Alita Burmeister (@AlitaBurmeister), at Yale University, USA, from work she did when working with Richard Lenski (@RELenski) at Michigan State University, USA. The paper, which also features one of our new Editors, Jenna Gallie (@gallie_jenna) from the Max Plank Institute for Evolutionary Biology in Plön, Germany, examines the evolutionary arms race between bacteria and bacteriophage, in this case *Escherichia coli* and phage lambda [4]. The paper exploits strains from a previous co-evolution experiment where samples had been taken at many points in the experiment, to go back and use whole-genome resequencing to reveal more about adaptations in the host and the virus during their prolonged co-evolution [5]. The paper, which was blogged about by the topic area lead, Mike Brockhurst (@BrockhurstLab), reveals additional complexity in the population dynamics of the host and the virus. Mutations that alter the LamB outer-membrane protein are well known to lead to resistance to lambda, as this is the initial receptor for the tail protein, but their study revealed that more was going on in the population as they saw the expansion of lines with mutants in genes encoding the ManYZ mannose phosphotransferase system in the inner membrane, which then later were lost in the population as the virus evolved new mutants to bypass this receptor and presumably latch onto another one. While the function of this sugar transport system as the inner-membrane receptor for lambda has been known since the 1970s [6], the population dynamics of changes in the both the outer membrane and inner membrane has never been observed before. It will be fascinating to find out how lambda has evolved to bypass ManYZ and what other receptor it now uses.

The precise role of ManYZ in this and other processes also suggests that the cell uses it for functions beyond a simple sugar transporter. The small protein DicB encoded by *E. coli* K-12 Qin prophage can inhibit ManYZ function and confer resistance to lambda infection, as recently discovered by Kari Vanderpool’s laboratory (@MicroPhysIL) at the University of Illinois, USA in 2019 [7]. However, this inhibition appears to be indirect, through DicB interacting with the cell division protein MinC and preventing its localization to the midcell. Strains with mutations in *minC* that abolish its interaction with DicB now lose the ability of DicB to inhibit ManYZ function. Clearly this is hinting at broader questions about the connection of this sugar transporter to the cell division apparatus. The general components of the PTS system, namely enzyme I (EI) and the HPr protein, have been shown to localize at the cell poles [8] and more recently the Bramkamp group with Gerd Seibold showed that the enzyme II components from multiple PTS in *Corynebacterium glutamicum* form clusters in the cell membrane [9]. All these studies point to higher organization of membrane transporter function in the cell membrane than we currently understand.

Before we move on, a quick mention of Richard Lenski, the senior author on the paper and the father of the *E. coli* long-term evolution experiment (LTEE). Being fascinated with this study as a graduate student working in *E. coli* respiration in the late 1990s, I wrote to Professor Lenski asking if he could send me a copy of one of his recent papers and received a few weeks later a US Air Mail envelope containing a whole pile of reprints and a kind note from Richard that I devoured over the next few weeks. Twenty years or so later the experiment continues, an incredible testimony to Richard’s dedication and prescience that these frozen samples of *E. coli* give microbiologists their own archaeological record to extract fundamental information about evolution. Although Darwin himself did not exemplify his theories with examples from microbiology, he would, I have no doubt, have been an enormous supporter and advocate of the LTEE.

The next article that took my eye was from the group of *Microbiology* Editor Kelli Palmer (@palmer_lab) at the University of Texas, Dallas, USA, continuing the publications of graduate student Luke Joyce (@Luke_Boitjie) [10]. The authors are interested in the dynamics of the membrane lipid composition of pathogenic streptococci, the investigation of which through lipidomics is still one of final frontiers of fundamental cell biology outside of model bacteria. Luke, who is now a postdoc in Kelly Doran’s laboratory (@KdoranLab) at the University of Colorado, had already discovered that *Streptococcus pneumoniae* only contained phosphatidylcholine (PC) in its membrane when cultured in the presence of glycerophosphatidylcholine (GPC), which is found in both the rich medium Todd–Hewitt broth and in the presence of human serum [11], suggesting that the bacterium can modify host-derived molecules for direct incorporation into the cytoplasmic membrane. In this study they expand the work to the other two major types of pathogenic streptococci, namely group A streptococci (GAS) and *Streptococcus agalactiae* (group B streptococci; GBS) and find that, as with *S. pneumoniae *, no PC is seen in the membrane in cells grown on minimal medium, but also not on the Todd–Hewitt media, although it was detected when human serum was added [10]. They surmise that these species can scavenge another form of PC called lyso-PC, which is known to be present in serum, and then modify this to add PC into their own membrane. This reminds me of work by the late Harry Smith FRS on the *in vivo* phenotype, as one of his major contributions to microbiology was the realization that in culture some pathogens actually had different cell surface virulence determinants compared to when directly isolated from patients [12]. This he exemplified by the discovery that pathogenic *Neisseria* had sialylyated lipopolysaccharide when isolated from patients, but not in culture, due to them directly incorporating this from host-derived CMP-sialic acid onto their own cell surface [13]. This direct addition of a host molecule led to serum resistance and protection from complement killing, as seen also in other pathogens such as *Haemophilus influenzae* that also scavenge sialic acid from the host to modify their own surface [14, 15]. It will be interesting to see the what the benefits are for the streptococci in the direct incorporation of PC into their membranes, rather than sticking with their ‘normal’ membrane composition and just using it as a nutrient. As well as this interesting scavenging of host molecules, they also detect the evidence of the plasmalogen version of PC in the membrane – this is where the fatty acyl chain of the lipid is attached to the head group by an ether bond rather than a normal ester bond. These lipids are normally associated with bacteria like *Clostridium* sp., where they are thought to confer protective effects on the membrane [16], and this is the first report of them occurring in these pathogens. Remarkably, the genes for introduction of these lipid modifications have only just been discovered [17, 18], illustrating how much more there is to do with bacterial lipidomics and how much more biology there is out there to discover and exploit.

Our next paper from Phillip Coburn (@PhillipSCoburn2) and colleagues, including Huzzatul Mursalin (@mdhmursalin), working in the laboratory of Michelle Callegan (@CalleganLab) at the University of Oklahoma, USA, concerns *Bacillus cereus* and its role in the serious eye infection, endophthalmitis. This disease, which occurs most frequently after eye operations, can be caused by several bacteria, including *Enterococcus faecalis *, *Staphylococcus aureus* and *S. pneumoniae *, getting into the interior of the eye to colonize the vitreous humour, and the resulting toxin production and inflammation can result in vision loss. In this paper the authors use a transcriptomics approach to study the differences in gene expression in *B. cereus* when grown on brain heart infusion (BHI) medium compared to when cultured in a murine endophthalmitis model [19]. They find a host of changes in expression patterns, some consistent with their existing knowledge, such as the upregulation of toxins *in vivo*, and massive upregulation of genes encoding superoxide dismutase enzymes. However, a known regulator *plcR* was not differentially expressed, suggesting that at the 8 h time point at which the *in vivo* sample was taken the cells were still growing rapidly as *plcR* is turned on by low nutrient availability and high cell density [20]. This single snapshot provides lots of new information about the *in vivo* environment that could now lead to larger studies looking at colonization and disease progression through longer periods of time to suggest the most suitable target processes to try and improve treatment of this disease.

Sticking with transcriptomic studies, we move away from pathogens to bacteria that are generally more beneficial for mankind, namely the ubiquitous producers of antibiotics that are the *Streptomyces *, and look at a paper from Vanessa Yoon Calvelo (@VYCalvelo) from the laboratory of Justin Nodwell (@JNodwell) at McMaster University, Canada, also featuring recent *Microbiology* Editor Marie Elliot. Their work studies the activation of genes for natural product formation in the model actinomycete *Streptomyces coelicolor* [21]. Getting these bacteria to produce antibiotics and other interesting chemicals that they would normally only form under specific environmental conditions is an ongoing area of research for many investigators and the work of *Microbiology* editor Matt Hutching manipulating the function of the two-component system MtrAB to activate numerous gene clusters comes to mind [22]. A different approach, however, is used in this new study, which is to understand how chemical elicitors that increase antibiotic production function. Here they study production of the blue pigmented antibiotic actinorhodin as their test system and use a transcriptomic approach to examine how the chemical ARC2 alters the physiology of *S. coelicolor *. In the presence of ARC2 they observed significant changes in genes involved in specialized metabolic clusters but also genes involved in primary metabolism. In amongst the upregulated genes they spotted two that were involved in the transcriptional regulation of antibiotic production, *afsS* and *afsR* [23], which they then studied further. Using a range of genetic methods, including a beautiful colour-based liquid culture assay to study complementation of actinorhodin production, they demonstrated that the ARC2 induction of actinorhodin production required both *afsR* and *afsS*, with AfsS being the final activator of actinorhodin production. They finally showed that constitutive expression of *afsS* alone can drive actinorhodin overexpression and concluded that its induction by ARC2 is a consequence of some stress response pathway(s) being activated that result in activation of AfsR and hence expression of *afsS*, suggesting that there are still gaps in our knowledge in the connection between the externally applied chemical and AfsR activation.

Deputy Editor in Chief Tracy Palmer and I were delighted to be able to tell our Editors and Senior Editors that submissions to *Microbiology* have increased in 2021 and we hope the #publishingforthecommunity message is getting out to authors as they chose where to send their papers.
